# Evaluation of the relationship between maxillary posterior teeth and the maxillary sinus floor using cone-beam computed tomography

**DOI:** 10.1186/s12903-018-0626-z

**Published:** 2018-10-03

**Authors:** Yechen Gu, Chao Sun, Daming Wu, Qingping Zhu, Diya Leng, Yang Zhou

**Affiliations:** 1 Department of Endodontics, Stomatological Hospital Affiliated to Soochow University, Suzhou Stomatological Hospital, Suzhou, Jiangsu People’s Republic of China; 2 Jiangsu Key Laboratory of Oral Diseases, the Affiliated Stomatological Hospital of Nanjing Medical University, 136 Hanzhong Road, Nanjing, 210029 People’s Republic of China; 3 Department of Oral Special Consultation, the Affiliated Stomatological Hospital of Nanjing Medical University, 136 Hanzhong Road, Nanjing, 210029 People’s Republic of China; 4 Department of Radiology, the Affiliated Stomatological Hospital of Nanjing Medical University, 136 Hanzhong Road, Nanjing, 210029 People’s Republic of China; 5 Department of Endodontics, the Affiliated Stomatological Hospital of Nanjing Medical University, 136 Hanzhong Road, Nanjing, 210029 People’s Republic of China

**Keywords:** Cone-beam computed tomography, Maxillary posterior teeth, Maxillary sinus floor, Root apices

## Abstract

**Background:**

Maxillary posterior teeth have close anatomical proximity to the maxillary sinus floor (MSF), and the race, gender, age, side and presence/absence of adjacent teeth may influence the mean distances between the root apices and the MSF. This study aimed to evaluate both the relationship between the maxillary posterior teeth and MSF, and the influence of adjacent teeth loss on the distance between the maxillary posterior roots and MSF.

**Methods:**

Cone-beam computed tomography images were collected from 1011 Chinese patients. The relationship between the maxillary posterior teeth and the MSF was divided into three types: Type OS (the root apex extending below/outside the MSF), Type CO (the root apex contacting with the MSF), Type IS (the root apex extending above/inside the MSF). The minimum vertical distances between the maxillary posterior roots apices and the MSF were recorded. The correlations of the distances with gender and age were analyzed. The distances between the maxillary posterior root apices and the MSF with different types of adjacent teeth loss was evaluated.

**Results:**

Type OS was the most common relationship of all posterior root apices (*P*<0.05). Type IS was highest in the palatal roots (PRs) of the maxillary first molars (MFMs) and the mesiobuccal roots (MBRs) of the maxillary second molars (MSMs) (24.8% and 21.6%) (*P*<0.05). The frequency of Type IS decreased with age except the premolar roots and PRs of the MSMs (*P*<0.05). The MBRs of the MSMs had the lowest distances to the MSF (0.8 ± 2.5 mm), followed by the distobuccal roots of the MSMs (1.3 ± 2.7 mm) and the PRs of the MFMs (1.4 ± 3.4 mm) (*P*<0.05). Age was an important influencing factor to the mean distances while gender had little effects. The distance between the maxillary second premolar root apices and the MSF decreased with the absence of adjacent teeth (*P*<0.05).

**Conclusions:**

The maxillary molars showed greater proximity to the MSF than premolars. Age had significant impacts on the relationship between maxillary posterior roots and MSF. The absence of maxillary first molars will influence the proximity of maxillary second premolar root apices to MSF.

## Background

The maxillary sinus (MS) is an important anatomic structure beside the nasal cavity and close to the root apices of maxillary posterior teeth. The maxillary sinus floor (MSF) is formed by the alveolar process of the maxilla and situated 5 mm inferior to the nasal floor at approximately 20 years old [1]. The inferior wall of sinus is lined with Schneiderian membrane which is changeable during the development of relevant disease [2]. Previous studies indicated that the volume of MS pneumatization is not a permanent state but a metabolic process, increasing at about 12 years old and reaching the lowest point at about 20 years old with the complete eruption of the maxillary third molars [3, 4]. In addition, the MS pneumatization can be affected by the extraction of the maxillary posterior teeth, especially several adjacent teeth, teeth with apices intruded into the sinus and maxillary second molars [1, 5, 6].

Due to the close anatomical proximity of the root apices of maxillary posterior teeth to the MSF, teeth infections may spread into the MS through periapical tissues and cause odontogenic maxillary sinusitis, which account for 10%~ 12% of all sinusitis [7]. The periapical and marginal lesion of roots close to or extending into the MSF could cause inflammatory changing of sinus mucosal lining and result in maxillary sinusitis in the end [8]. The infection can also intrude into the sinus via bone marrow, blood vessels and lymphatics. Mehra and Murad reported that when there is a close proximity of root apices of teeth with necrotic pulp and the MSF, the MS can also be influenced [9]. If clinical operation errors occur during root canal treatment, the root canal shaping instruments, flushing fluid and filling materials can extrude to the MS subsequently. In addition, a perforation of MSF can occur and result in oroantral fistula during tooth extraction, and a pathological change of MS can also occur for the improper implant therapy [10]. All these reasons can result in various complications, such as odontogenic maxillary sinusitis, endo-antral syndrome and traumatic alterations, which are complex problems for dentists and otolaryngologists [7, 11].

Periapical and panoramic radiographies are conventional imaging techniques used for treatment planning and evaluating the close relationship of the root apices and the MSF. However, they can only provide two-dimensional (2D) images, causing superposition and magnification of anatomic structures impending proper diagnosis. During periapical surgery, the 2D radiographies cannot determine the risk of perforation of MSF [12]. In past 20 years, cone-beam computed tomography (CBCT) has gradually got into the public eyes. As a three-dimensional (3D) imaging technique, CBCT has important significances in clinical diagnosis and planning process, contributing to the establishment of effective therapeutic protocols [13]. When compared with conventional CT, CBCT works with lower radiation, higher resolution and shorter scanning time [14]. CBCT can provide high-quality 3D images of oral and maxillofacial regions and evaluate the relationship between the maxillary root apices and MS clearly.

Previous studies evaluated the proximity of the maxillary posterior teeth to the MSF. For example, Jung et al. [15] reported that the mesiobuccal roots (MBRs) of the maxillary second molars (MSMs) were closest to the MSF in Korean people, but they did not analyze the correlations of the distances with gender and age. Von Arx et al. [16] evaluated the distances between maxillary premolar roots and the MSF in Switzerland population, and found that gender, age, side, and presence/absence of premolars failed to influence significantly the mean distances between premolar roots and the MS. Kilic et al. [17] and OK et al. [18] evaluated the relationship between the maxillary posterior teeth root tips and the MSF in Turkey population respectively, both of them found that there were no significant statistical differences between the right and left side measurements, but them had different conclusions in the relationships between males and females. In addition, OK et al. [18] also found that the relationship between the posterior teeth and the MSF differed according to the age decade interval. However, few studies have investigated the relationship between the root apices of maxillary posterior teeth and the MSF in Chinese population, and whether the gender, age, side and presence/absence of adjacent teeth influence the mean distances between the root apices and the MSF also unclear. Therefore,the aim of this study was to evaluate the anatomic proximity of the maxillary posterior roots apices to MSF in Chinese population according to age, gender and side. In addition, the distances between the root apices of maxillary posterior teeth and the MSF with different situation of adjacent teeth loss were also evaluated.

## Methods

### CBCT images collections

This study was approved by the Ethical Committee Department, the Affiliated Stomatological Hospital of Nanjing Medical University (PJ2015–047-001), and the written informed consent was obtained from the patients. High-quality CBCT images were randomly collected from the Department of Radiology, the Affiliated Stomatological Hospital of Nanjing Medical University from May 2017 to October 2017.

The reasons for CBCT scanning include dental implants, endodontic treatment, treatment planning before orthodontics and diagnosis of facial trauma. The CBCT images showed the maxillary premolars, molars and MSF clearly. The exclusion criteria for the CBCT images were as following: (1) Maxillary premolars or molars with caries, unformed apices, root resorption or fractures. (2) Maxillary premolars or molars with root canal fillings, posts or coronal restorations. (3) The presence of periapical or periradicular lesions, or MS diseases. (4) Patients have been experienced orthodontic treatment, or experienced traumatic injury which disturbing the normal anatomy of MS.

CBCT images of 1745 maxillary first premolars (MFPs), 1663 maxillary second premolars (MSPs), 1331 maxillary first molars (MFMs) and 1360 MSMs from 1011 individuals (476 males and 535 females) were selected. The age of these patients was ranged from 18 to 85 years, with mean age of 47.7 ± 15.6 years. The patients were divided into 3 groups: 18–40 years group, 41–60 years group and ≥ 61 years group.

### CBCT images evaluations

CBCT images were obtained using a CBCT scanner (NewTom VG, QR srl., Verona, Italy) with the following parameters:110kVp, 3.6–4.8 mA, a voxel size of 0.2 mm and a field of view of 12 × 8 cm or 15 × 15 cm. The as-low-as-reasonably achievable principle was strictly followed, exposing patients to the least amount of radiation while still obtaining the most useful information for a proper diagnosis. All images were acquired by an experienced radiologist according to the manufacturer’s instructions.

**Fig. 1:**
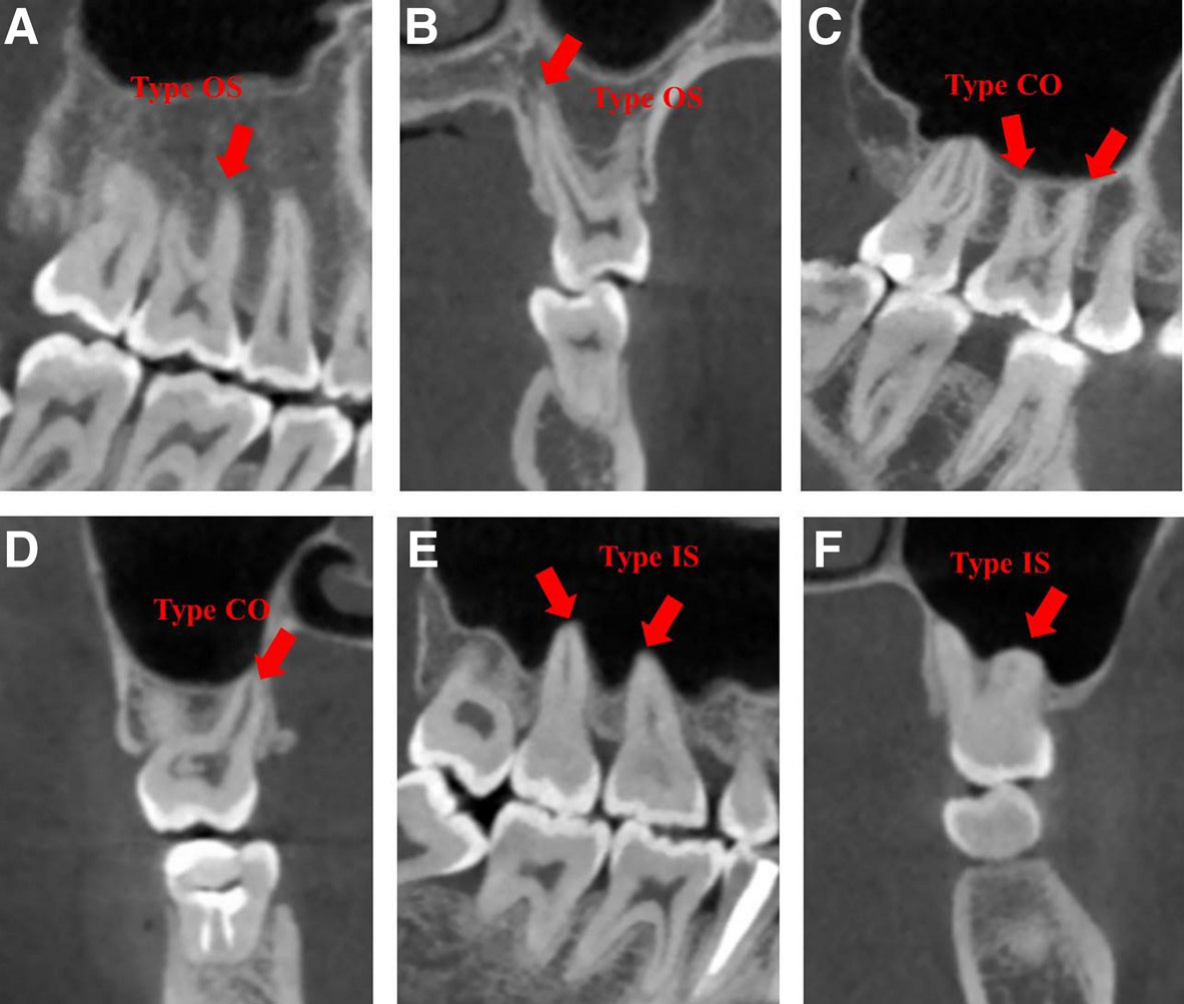
CBCT images of three vertical relationships between maxillary posterior teeth and MSFs in sagittal (**a**, **c** and **e**) and coronal planes (**b**, **d** and **f**). **a** Type OS: MBR of a right MFM; **b** Type OS: PR of a left MFM; **c** Type CO: MBR and DBR of a right MFM; **d** Type CO: PR of a right MSM; **e** Type IS: PRs of right maxillary molars; **f** Type IS: MBR of a left MSM

To ensure the reliability of the values, two endodontists calibrated their interpretations by reviewing 20 CBCT images of MSF and maxillary posterior teeth selected before the experimental reading. The intra and inter-examiner reliability were assessed by Cohen’s kappa statistical analysis. They assessed the CBCT images with NNT4.6 software (QR srl., Verona, Italy) simultaneously. The software reorientation was performed parallel to the occlusal surface before measuring in reconstruction. The contrast and brightness of the images could be adjusted using the software to ensure optimal visualization. The vertical relationship between the maxillary posterior root apices and the MSF were evaluated in CBCT sagittal and coronal planes simultaneously (Fig. 1): (1) Type OS: the root apex extending below/outside the MSF; (2) Type CO: the root apex contacting with the MSF; (3) Type IS: the root apex extending above/inside the MSF.

**Fig. 2:**
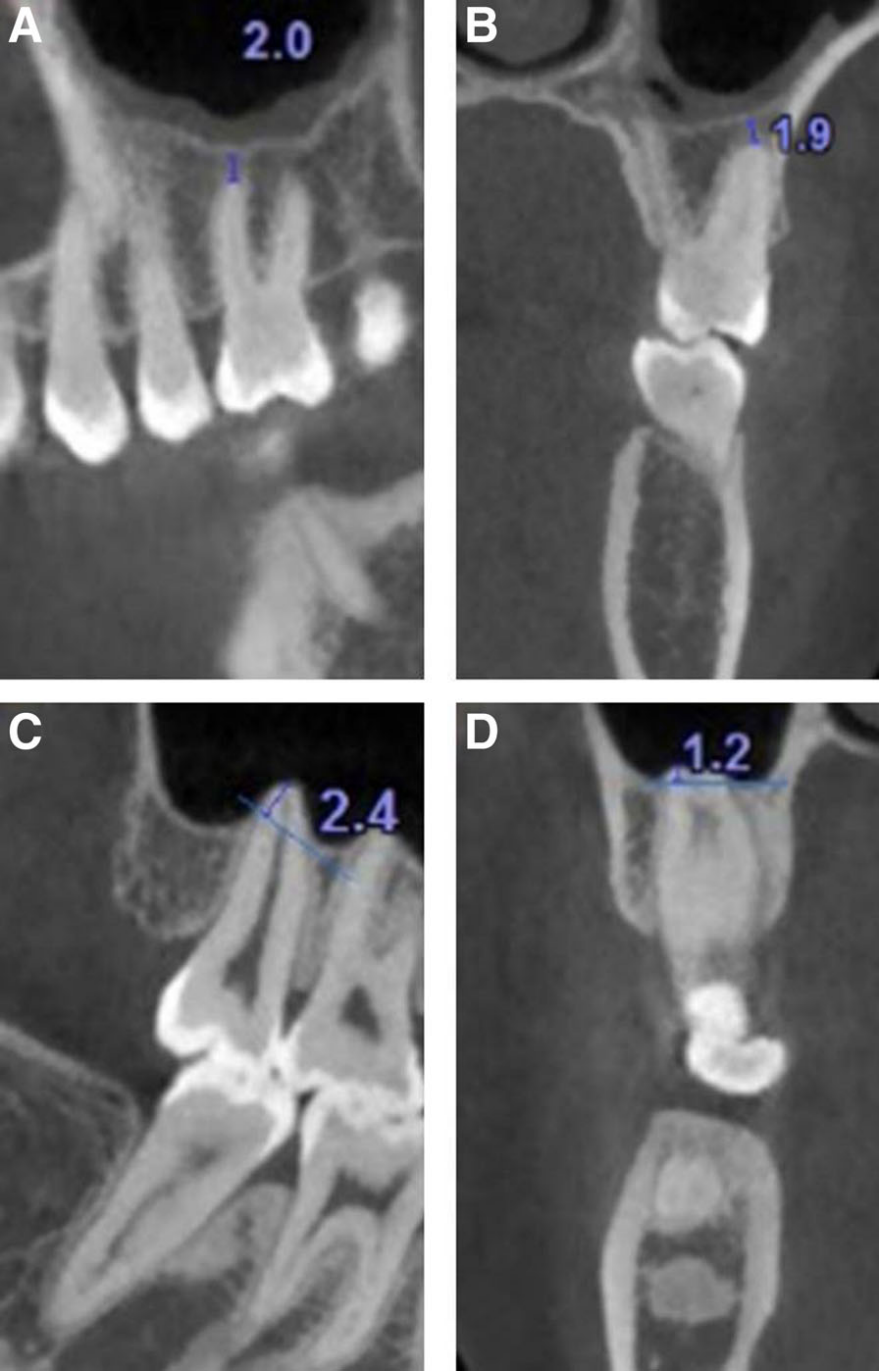
The shortest distances between the root apices and the MSFs were measured. **a** CBCT sagittal plane, positive value; **b** CBCT coronal plane, positive value; **c** CBCT sagittal plane, negative value; **d** CBCT coronal plane, negative value

The vertical distance of the maxillary posterior root apices to the MSF was measured according to previous studies [19]. Briefly, the vertical distance between the root apices of each maxillary posterior tooth and the closest border of the MSF was measured in serial sagittal and coronal planes with a slice thickness of 0.2 mm. The shortest distance was recorded. A negative value was recorded if the root apex intruded into the MSF. Only one value was recorded if the roots were fused (Fig. 2). The correlations of the vertical relationship and distance with age and gender were analyzed.

In order to evaluated the changes in the distances of root apices of maxillary posterior teeth to MSF in right and left side with different situation of adjacent teeth loss, the CBCT images of the patients who experienced one side tooth extraction more than 6 months were assessed. Then the vertical distance of the side that with tooth extraction were compared with the other side that without tooth extraction.

### Statistical analysis

The statistical analysis was performed using the SPSS 22.0 (IBM Corp., Armonk, NY, USA) at a significance level of *P*<0.05. The association between the distance measurements and the patient’s age, gender and side were assessed using the chi-square test and the Kruskal-Wallis test. The Wilcoxon test was used to analyze the influence of adjacent teeth loss.

## Results

The kappa values for the intra-examiner agreements of each examiner were 0.923 and 0.933, respectively. Regarding inter-examiner agreement, the kappa values were 0.898 and 0.877 for the first and second assessments, respectively. There was a good intra-examiner and inter-examiner agreement.

### The vertical relationship between the maxillary posterior root apices and the MSF

The distribution of the relationship between maxillary posterior root apices and the MSF were shown in Tables 1 and 2. Type OS was the most common relationship of all root apices to the MSF, and it was the highest in the MFPs (*P*<0.05). Type IS was highest in the palatal roots (PRs) of the MFMs and the MBRs of the MSMs (24.8% and 21.6%). The frequency of Type IS decreased with age except the premolar roots and PRs of the MSMs (*P*<0.05) .

**Table 1:** Distribution of the relationship between maxillary premolars root apices and the MSFs by age (%)

Age	MFPs			MSPs		
	Type OS	Type CO	Type IS	Type OS	Type CO	Type IS
18-40y	85.2	10.8	4	52	32.3	15.7
41-60y	95	5	0	71	25.8	3.2
>60y	97.5	2.5	0	81.7	13.9	4.4

**Table 2:** Distribution of the relationship between maxillary molars root apices and the MSFs by age (%)

Age	MBR			DBR			PR		
	Type OS	Type CO	Type IS	Type OS	Type CO	Type IS	Type OS	Type CO	Type IS
MFMs 18-40y	41.5	36.8	21.7	40.4	37.9	21.7	37.7	25.7	36.6
MFMs 41-60y	61.5	30.7	7.8	62.3	31.7	6.0	52.8	30.1	17.1
MFMs >60y	74.6	22.6	2.8	76.0	21.2	2.8	63.2	27.6	9.2
MSMs 18-40y	29.5	35.1	35.4	36.8	39.0	24.2	44.2	35.7	20.1
MSMs 41-60y	48.7	40.4	10.9	60.0	31.6	8.4	65.7	28.2	6.1
MSMs >60y	54.0	37.4	8.6	66.7	26.5	6.8	66.7	25.2	8.1

There were no significant differences between males and females in the occurrences of Type IS in all posterior roots (*P*>0.05). There were no significant differences between two sides as well (*P*>0.05).

### The distances between the maxillary posterior root apices and the MSF

There was no significant differences between right and left side maxillary posterior root apices of the distances (*P*>0.05).

The MFPs had the largest distances, while the MBRs of the MSMs had the lowest distances for both males and females, followed by the distobuccal roots (DBRs) of the maxillary second molars and the PRs of the maxillary first molars (*P*<0.05). There were no significant differences in the mean distance between males and females (*P*>0.05) (Table 3).

**Table 3:** The shortest vertical distances between the maxillary posterior root apices and the MSFs according to sex ($\overline{x}$ ± SD, mm)

	MFPs	MSPs	MFMs			MSMs			*P*
			MBR	DBR	PR	MBR	DBR	PR	
Male	6.0 ± 4.4	2.5 ± 3.6	1.9 ± 3.3	1.8 ± 3.1	1.3 ± 3.2	0.7 ± 2.4	1.2 ± 2.6	1.8 ± 3.0	
Female	5.8 ± 4.4	2.6 ± 3.6	2.0 ± 3.4	1.9 ± 3.3	1.5 ± 3.5	0.8 ± 2.7	1.4 ± 2.8	2.2 ± 3.4	>.05
Total	5.9 ± 4.4	2.5 ± 3.6	1.9 ± 3.3	1.9 ± 3.1	1.4 ± 3.4	0.8 ± 2.5	1.3 ± 2.7	2.0 ± 3.2	

The distances between the root apices and the MSF increase with age in all roots of posterior teeth and there were significant differences among groups (*P*<0.05) (Table 4).

**Table 4:** The shortest vertical distances between the maxillary posterior root apices and the MSFs according to age ($\overline{x}$ ± SD, mm)

Age	MFPs	MSPs	MFMs			MSMs			*P*
			MBR	DBR	PR	MBR	DBR	PR	
18-40y	4.9 ± 3.9	1.7 ± 3.0	1.2 ± 2.9	1.2 ± 2.8	0.8 ± 3.1	0.2 ± 2.3	0.8 ± 2.4	1.3 ± 2.7	.00*
41-60y	6.8 ± 4.3	3.4 ± 3.8	2.6 ± 3.3	2.6 ± 3.2	2.1 ± 3.5	1.5 ± 2.7	2.1 ± 3.0	2.8 ± 3.5	
≥61y	8.3 ± 4.6	4.6 ± 4.0	3.8 ± 3.9	3.7 ± 3.8	3.0 ± 3.9	2.2 ± 3.2	2.9 ± 3.4	3.5 ± 3.9	

*The distances between the root apices and the MSFs increased with age and there were significant differences among the groups

### The distance between the root apices and the MSF influenced by loss of adjacent teeth

The distance between root apices of the MSPs and the MSF was significantly shorter with loss of adjacent teeth (Tables 5 and 6).

**Table 5:** Comparison of the vertical distances of root apices of maxillary premolars with/without adjacent teeth ($\overline{x}$ ± SD, mm)

	BR/PR	*P*
MFPs A	4.7 ± 3.6	.31
MFPs P	5.0 ± 3.9	
MSPs A	2.6 ± 2.6	.01*
MSPs P	1.9 ± 2.3	

*A/P* Absence/Presence of adjacent teeth

* There were significant differences in 2 PM with or without absence of adjacent teeth

**Table 6:** Comparison of the vertical distances of root apices of the maxillary molars with/without adjacent teeth ($\overline{x}$ ± SD, mm)

	MBR	DBR	PR	*P*
MFMs A	2.6 ± 2.3	1.8 ± 2.2	1.5 ± 2.4	.41
MFMs P	2.0 ± 2.4	2.0 ± 2.8	1.8 ± 2.9	
MSMs A	0.7 ± 1.2	1.1 ± 1.4	1.6 ± 1.9	.66
MSMs P	0.8 ± 1.5	1.4 ± 1.5	1.9 ± 2.2	

*A/P* Absence/Presence of adjacent teeth

## Discussion

The close anatomic distance between the maxillary posterior root apices and the MSF result in various complications during disease development and dental treatment process. In present study, the proximity of the maxillary posterior root apices to the MSF in a native Chinese population was studied. The results showed that the Type OS (the root apex extending below/outside the MSF) was observed in 91.9% of MFPs, which is consistent with previous studies [17, 20, 21], indicating that the roots of MFPs have little relationship with the MSF. For MSPs, the frequencies of Type IS (the root apex extending above/inside the MSF) were observed a few times as many as the MFPs and the Type OS accounted for 66.0%, which showed that the relationship of the MSPs is relatively close to the MSF. There were no significant differences between the left and right premolars, which is also in agreement with the findings of Kilic et al. [17] and OK et al. [18]. For maxillary molars, the Type IS was observed more frequently in the PRs of the MFMs (24.8%) and the MBRs of the MSMs (21.6%), indicating that dentists should pay more attentions to the two roots during the dental treatment, because the perforation of MSF in the areas is more likely to occur. However, Pagin et al. [20] reported that the root protrusions (Type IS) in Brazilian population were 3.2%, 1.8% and 5.5% for MBRs, DBRs and PRs of MFMs, 12.9%, 8.3% and 4.1% for MBRs, DBRs and PRs of the MSMs. Jung and Cho [15] reported that the root protrusions in Korean population were 32.5% and 30.1% for MBRs and DBRs of MFMs, and 36.7% and 34.3% for MBRs and DBRs of MSMs. OK et al. [18] reported that the root protrusions in Turkey population were 34.2% for MBRs of MFMs and 30.9% for MBRs and DBRs of MSMs. These results were different with the present study. Possible explanation is that ethnic difference is an important factor to influence the relationship between the maxillary posterior root apices and the MSF.

The MS pneumatization varied with age [1, 3]. Kalender et al. [22] reported that the lateral recess of the MS is more likely to interpose between roots in people aged 18–54 years compared to those aged 55 years and older in Turkish population. Tian et al. [21] investigated the mean distances between posterior root apices and the MSF in Chinese population, and they found that the mean distances to the MFS increased and the frequencies of Type IS decreased with age. Considering that the Type IS was closely related to the development of odontogenic maxillary sinusitis, so the present experiment focused on the Type IS proportion in Chinese population. The results showed that the frequency of Type IS decreased with age for all maxillary posterior roots. Therefore, age is the important factor to influence the relationship between the maxillary posterior root apices and the MSF.

The results of this study showed that the root apices of MFPs had the maximum distance to the MSF (5.9 ± 4.4 mm), which were consistent with the previous reports in Turkey and Brazilian populations [17, 19], indicating that the inadequate endodontic treatments of the MFPs make less impacts on the condition of the MS mucosa when compared with the maxillary molars because of a closer anatomic relationship between maxillary molars and the sinus floor [23]. It was interest to find that the MBRs of MSMs had the minimum distance to the MSF (0.8 ± 2.5 mm),which was in agreement with the previous studies using CBCT technology in Romania and Chinese populations [21, 24]. However, Yoshimine et al. [25] reported that the PRs of the MFMs had the shortest distance to the MSF (1.67 ± 2.36 mm) in Japanese population. Kilic et al. [17] and Kwak et al. [26] found that the shortest distance was observed in DBRs of the MFMs (2.74 ± 3.23 mm and 0.25 ± 2.17 mm, respectively) in Korean and Turkey populations. Ethnic and different measurement methods may influence the distances between the maxillary posterior root apices and the MSF. In this study, the distances between all roots and the MSF increased with age, indicating that younger people had more probability to have odontogenic maxillary sinusitis caused by iatrogenic factors. However, the proportion of periodontitis and periapical periodontitis is also higher among the elderly, so it is not possible to infer that the incidence of odontogenic maxillary sinusitis in the elderly is lower than the young people.

Tooth extraction can influence the location of the MSF [1]. von Arx et al. [16] measured the distances of the MFPs with or without the presence of MSPs and found the distances tended to be greater with the presence of MSPs, but the results didn’t have significant differences. In this study, the changes of vertical relationship between the maxillary posterior root apices and the MSF after the extraction of adjacent teeth were evaluated using the self-control study. The results showed that when the MFMs or the MFPs was absent, the distances between the roots of the MSPs and the MSF decreased significantly, while the other roots had no significant effect. Compared with premolars, MFMs were closer to the MSF and had relatively more obvious impact on the reconstruction of the MSFs. Therefore, it can be inferred that the loss of the MFMs may lead to a forward expansion to the maxillary sinus and influence the distances of MSPs to the MSF.

## Conclusions

With the limitations of this study, it may be concluded that the MBRs of MSMs appeared to be the closest to the MSF. The frequency of root apices extending outside the MSF increased with age, while the frequency of the root apices extending inside or contacting with the MSF decreased with age. The absence of the MFMs could influence the proximity of the MSPs roots to the MSF.

## Abbreviations

2D: Two-dimensional
3D: Three-dimensional
CBCT: Cone-beam computed tomography
DBRs: Distobuccal roots
MBRs: Mesiobuccal roots
MFMs: Maxillary first molars
MFPs: Maxillary first premolars
MS: Maxillary sinus
MSF: Maxillary sinus floor
MSMs: Maxillary second molars
MSPs: Maxillary second premolars
PRs: Palatal roots
